# Comparison of Inflammation-Based Prognostic Scores in a Cohort of Patients with Resectable Esophageal Cancer

**DOI:** 10.1155/2017/1678584

**Published:** 2017-06-27

**Authors:** G. Jomrich, M. Paireder, A. Gleiss, I. Kristo, L. Harpain, S. F. Schoppmann

**Affiliations:** ^1^Department of Surgery, Medical University of Vienna and Gastroesophageal Tumor Unit, Comprehensive Cancer Center (CCC), Spitalgasse 23, 1090 Vienna, Austria; ^2^Center for Medical Statistics, Informatics, and Intelligent Systems, Medical University of Vienna, Spitalgasse 23, 1090 Vienna, Austria

## Abstract

**Background:**

A number of studies have revealed that inflammation-based prognostic scores, such as Glasgow prognostic score (GPS), modified Glasgow prognostic score (mGPS), and C-reactive protein and albumin ratio (C/A ratio), are associated with poor outcome in cancer patients. However, until now, no study has investigated the role of these prognostic scores in a cohort of neoadjuvant-treated esophageal adenocarcinomas (nEAC) and squamous cell carcinomas (nESCC).

**Methods:**

Patients had laboratory measurements within three days before resection. GPS, mGPS, and C/A ratio were tested together with established clinicopathological factors in simple and multiple Cox regression analysis of overall survival (OS) and disease-free survival (DFS).

**Results:**

A total of 283 patients (201 EAC and 82 ESCC) with locally advanced esophageal cancer were enrolled. 167 patients received neoadjuvant treatment (59.0%). Simple analysis revealed that there were significant differences in cancer-specific survival in relation to elevated C-reactive protein (*p* = 0.011), lymph node status (*p* < 0.001), UICC stage (*p* < 0.001), and nEAC (*p* = 0.005). mGPS (*p* = 0.024) showed statistical significance in simple analysis. No statistical significance could be found for GPS (*p* = 0.29), mGPS (*p* = 0.16), and C/A ratio (*p* = 0.76) in multiple analysis.

**Conclusion:**

The investigated prognostic scores should be used and interpreted carefully, and established factors like histology, including tumor size and differentiation, lymph node involvement, and status of resection margin remain the only reliable prognostic factors for patients suffering from resectable EC.

## 1. Introduction

With an estimated 456.000 new cases in 2012, esophageal cancer (EC) is the eighth most common cancer worldwide. Whereas the number of esophageal squamous cell carcinoma (ESCC) is decreasing, the number of esophageal adenocarcinoma (EAC) is increasing dramatically [1]. Combination of neoadjuvant chemoradio therapy (NCRT) and surgical resection has become the standard in treatment of locally advanced EC [2, 3]. Even though improvements in diagnosis, surgical techniques, and multidisciplinary therapeutical approaches of EC can be noticed, prognosis for patients after resection remains poor with a 3- and 5-year overall survival of 44% and 26%, respectively [4]. After resection of EC, prognosis has been found to be dependent on different factors, such as histology, including tumor size and differentiation, lymph node involvement, and status of resection margin [5–12]. Most of these factors are determined after surgery only. Therefore, it is useful to evaluate potential prognostic factors, which are available preoperatively.

In tumorigenesis, inflammation plays a crucial role. Due to an inflammatory microenvironment, tumor cell proliferation and migration as well as invasion, metastasis, cell survival, and neoangiogenesis is promoted [13, 14].

Previous studies investigated that C-reactive protein (CRP) and albumin levels may represent potential prognostic markers in various cancers [15–20].

Recent studies have shown inflammation-based prognostic scores, including Glasgow prognostic score (GPS), modified Glasgow prognostic score (mGPS), and CRP/Albumin (C/A) ratio to be a significant prognostic indicator in many cancers, including EC [21–29].

To our knowledge, the C/A ratio has not been investigated in a patient collective consisting of both, esophageal squamous cell carcinoma (ESCC) and esophageal adenocarcinoma (EAC). In this underlying study, in order to identify parameters to select patients who will have a poor prognosis after curative surgery, we evaluated and compared the prognostic role of inflammatory biomarkers, including the prognostic scores GPS, mGPS, and C/A ratio in a cohort of resectable EC. Additionally, we examined and compared inflammatory biomarkers and prognostic scores in a subset of EC patients who received neoadjuvant treatment prior to surgical resection.

## 2. Material and Methods

### 2.1. Patients

Medical records of 449 patients with histologically proven esophageal carcinoma (EC) were reviewed retrospectively. All patients received surgical resection of the esophagus between January 2003 and December 2014, at the Department of Surgery of the Medical University Vienna. The study was approved by the Ethics Committee of the Medical University of Vienna, Austria, according to the declaration of Helsinki. All blood samples were obtained within three days before surgery in line with standard preoperative procedures. Data regarding potential factors were assembled from medical records, including patients' age and sex, preoperative levels of serum CRP and albumin, tumor location, size, stage according to the 7th edition of the Union for International Cancer Control (UICC) and TNM staging according to the American Joint Committee on Cancer (AJCC) [30], tumor differentiation, resection margin, presence of lymphatic invasion, date and kind of surgical procedure, and NCRT.

Depending on histological type of cancer and preoperative treatment, four patient collectives were defined: nEAC (neoadjuvant-treated esophageal adenocarcinoma), EAC (preoperative untreated esophageal adenocarcinoma), nESCC (neoadjuvant-treated esophageal squamous cell carcinoma), and ESCC (preoperative untreated esophageal squamous cell carcinoma).

Patients who had recently pyrexia (axillary ≥ 37.2°C/99.0°F), any form of active infection or chronic inflammatory disease as well as patients with distant metastasis at time of presentation were excluded. All patients underwent regular, outpatient follow-up at the Medical University of Vienna.

CRP levels were determined by particle-enhanced immunoturbidimetry, and albumin was quantified by means of colorimetry using bromocresol green (depending on the date of blood testing: Olympus, Tokyo, Japan; Beckman Coulter, Brea, USA; Roche Diagnostics, Rotkreuz, Switzerland) under controlled conditions at the Department of Laboratory Medicine, Medical University of Vienna, which runs as the central laboratory of the General Hospital of Vienna a certified (ISO 9001) and accredited (ISO 15189, since 2008) quality management system [31].

Based on the laboratory standards of the Medical University of Vienna, CRP levels of >1 mg/dl were considered as elevated and albumin levels of <35 g/l were considered as hypoalbuminemia. GPS was constructed as previously described [32–34]. In brief, patients with both an elevated CRP (>1 mg/dl) and hypoalbuminemia (<35 g/l) were given a score of 2 (GPS2). Patients with neither of these abnormalities were scored as 0 (GPS0), and patients who had abnormal values for only one of the biochemical parameters were given score 1 (GPS1). The use of the mGPS was proposed by previous studies [35, 36]. Depending on the absence or presence of hypoalbuminemia, patients with CRP levels >1 mg/dl were given a score of 1 or 2 (mGPS1 or mGPS2). Patients with any level of albumin and a normal CRP were scored as 0 (mGPS0). C/A ratio was calculated as described previously by dividing serum CRP by the level of serum albumin [37].

Overall survival (OS) was defined as the time between primary surgery and the patients' death. Deaths from another cause than EC or survivals until the end of the observation period were considered as censored observations. Disease-free survival (DFS) was defined from the day of surgery until first evidence of disease progression.

## 3. Statistical Analysis

Continuous baseline characteristics are described with means and standard deviations (SD) in case of approximate normal distribution and with medians and quartiles otherwise. Categorical variables are described by counts and percentages. The probability distributions of overall survival (OS) and disease-free survival (DFS) are described by Kaplan-Meier curves. Median follow-up time is calculated using the inverse Kaplan-Meier method [38].

Simple and multiple Cox proportional hazards regression models are used to quantify the unadjusted and adjusted effects of various potential predictors on survival. Continuous predictors are investigated for nonlinear or time-dependent effects. No significant nonlinear effects were detected. The hazard ratios (HR) for time-dependent effects are evaluated at the clinically relevant time of 48 months after surgery. Lymph node status is represented with one indicator for at least one positive lymph node and another indicator for at least three lymph nodes (further distinction did not prove statistically significant). The number of resected lymph nodes was transformed using a binary log transformation such that the corresponding HR quantifies the effect of a doubling of this number.

Two interactions were preselected for analysis: the interaction of the indicator distinguishing nEAC from EAC with the indicator of nESCC versus ESCC and the interaction of preoperative elevated CRP and preoperative hypoalbuminemia. In case of significance, these are included in the model and HRs for one variable is given separately for categories of the other.

The relative importance of the investigated predictors of survival is quantified using marginal and partial proportions of explained variation (PEV) [39].

All calculations were performed using SAS 9.4 (SAS Institute Inc., Cary, NC, USA, 2012). Two-sided *p* values ≤ 0.05 are regarded as indicating statistical significance.

## 4. Results

### 4.1. Demographic Characteristics

From 449 patients enrolled, data from 283 patients was available for further investigation. The majority of the patients were males (*n* = 225, 79.5%), more than half received neoadjuvant treatment (*n* = 167, 59.0%). Mean age at diagnosis was 63 years (SD 10.4, range 31–88 years), with 30-day survival rate of 96.5% (95% CI, 93.5–98.1%).

Among these, majority of patients presented with UICC stage III at time of surgery (*n* = 97, 34.3%) and underwent two-stage surgery (*n* = 233, 82.3%), and two hundred fifty-three (89.4%) patients did not receive adjuvant therapy. Clinicopathological baseline characteristics are shown in Table 1.

Preoperative elevated CRP was found in 54 (19.4%) patients and hypoalbuminemia in 32 (11.4%) patients. From 4 patients, no preoperative CRP and/or albumin values were available. The majority of patients presented with a score of 0 for all prognostic scores evaluated (GPS 0 (*n* = 207, 74.2%); mGPS 0 (*n* = 225, 80.7%); C/A ratio 0 (*n* = 262, 93.9%)).

Details for subgroups nEAC, EAC, nESCC, and ESCC are summarized in Table 2.

The median follow-up time was 63 months (lower quartile 35, upper quartile 95 months).

Median OS of 283 patients eligible was 42.4 months (lower quartile 13.9 months, upper quartile not reached within period of observation), and 155 patients died during follow-up period. Elevated preoperative CRP (HR 1.63; 95% CI, 1.12–2.38; *p* = 0.011), lymph node status N1 and N2 (HR 3.42; 95% CI, 2.01–5.82; *p* < 0.001; and HR 3.13; 95% CI, 2.21–4.43; *p* < 0.001), advanced UICC stage (HR 2.90; 95% CI, 1.45–5.84; *p* = 0.003), neoadjuvant treatment in EAC (HR 1.85; 95% CI, 1.21–2.84; *p* = 0.005), and one-stage surgery (HR 0.56; 95% CI, 0.33–0.94; *p* = 0.027) were significantly associated with OS using simple Cox models for analysis. No significant association in simple analysis was found for preoperative hypoalbuminemia (HR 1.01; 95% CI, 0.61–1.68; *p* = 0.97), number of lymph nodes resected (HR 0.90; 95% CI, 0.77–1.05; *p* = 0.18), and neoadjuvant treatment in ESCC (HR 0.82; 95% CI, 0.78–1.42; *p* = 0.48). From all preoperatively analyzed scores, only mGPS (*p* = 0.024) showed statistical significance in simple analysis.

Lymph node status (PEV 12.4%) and UICC stage (PEV 13.5%) exhibit the largest proportions of explained variation in overall survival, whereas elevated preoperative CRP explains only 1.6%.

The multivariable analysis using a multiple Cox proportional hazards model revealed that lymph node status N1 and N2 (HR 1.93; 95% CI, 0.98–3.77; *p* = 0.007; and HR 2.37; 95% CI, 1.5–3.74; *p* < 0.001), number of resected lymph nodes (HR 0.7; 95% CI, 0.59–0.83; *p* < 0.001), and advanced UICC stage (HR 2.58; 95% CI, 1.08–6.14; *p* = 0.033) are independent prognostic factors. Advanced UICC stage (PEV = 3.0), number of resected lymph nodes (PEV = 3.2), and lymph node status N2 (PEV = 2.6) exhibit the largest proportions of explained variations in overall survival, whereas lymph node status N1 explains only <0.1% (Table 3).

No statistical significance regarding OS could be found in the multiple Cox proportional hazards model for elevated preoperative CRP (HR 1.47; 95% CI, 0.97–2.24; *p* = 0.07) and preoperative hypoalbuminemia (HR 0.92; 95% CI, 0.54–1.58; *p* = 0.76). Furthermore, our previously proposed ways to summarize CRP and albumin in to prognostic scores did not show significant effect on overall survival (GPS, *p* = 0.29; mGPS, *p* = 0.16; C/A ratio, *p* = 0.76). Accordingly, the PEVs are below 1% for GPS, mGPS, and C/A ratio. No statistical significance regarding OS and DFS could be found for the factors age and sex (*p* > 0.05, resp.; data not shown) in the multiple Cox proportional hazards model (Tables 3 and 4).

The median DFS of 266 patients eligible was 31 months (lower quartile 30, upper quartile 7 months), and 150 patients developed tumor recurrence during follow-up period. Regarding DFS, the factors lymph node status N1 and N2 (*p* = 0.001 and *p* < 0.001, resp.), neoadjuvant treatment in EAC (*p* = 0.002), and advanced UICC stage (*p* < 0.001) were significantly associated in simple analysis, whereas significant association could be found for lymph node status N2 (HR 2.58; 95% CI, 1.63–4.10; *p* < 0.001), number of lymph nodes resected (HR 0.78; 95% CI, 0.65–0.93, *p* = 0.005), and advanced UICC stages II and III/IV (HR 2.27; 95% CI, 1.04–4.95, *p* = 0.04 and HR 3.43; 95% CI, 1.42–8.33, *p* = 0.006). No significant correlation for preoperative CRP, preoperative hypoalbuminemia, and the prognostic scores GPS, mGPS, and C/A ratio and DFS could be found in simple and multiple analysis. Details can be found in Tables 5 and 6.

**Figure 1:**
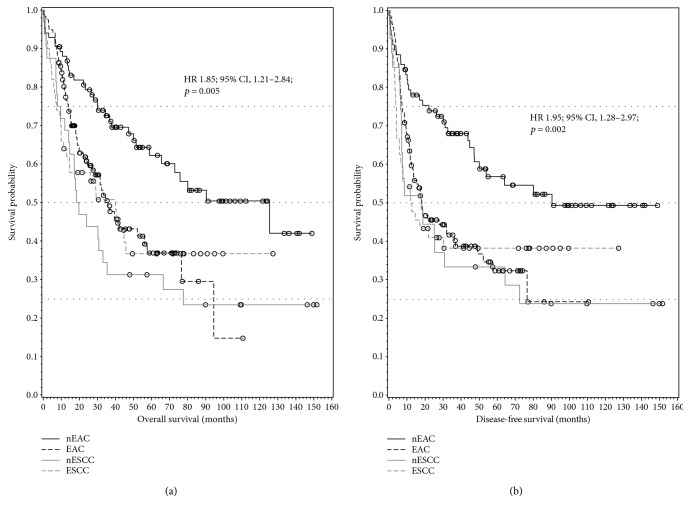
(a, b) Kaplan-Meier plots showing significantly better OS (overall survival) and DFS (disease-free survival) in nEAC (neoadjuvant-treated) patients, respectively.

Kaplan-Meier curves for the probability distribution of OS and DSF in the subgroups EAC, nEAC, ESCC, and nESCC are shown in Figure 1. The results of the simple Cox models revealed a statistically significant interaction between neoadjuvant therapy and the indicator for EAC versus ESCC (this interaction is not significant in the multiple Cox model nor is neoadjuvant therapy altogether). This means that, without adjustment for further variables, the effect of neoadjuvant therapy is different between EAC and ESCC (Table 3). Patients with nEAC showed a significantly better OS (HR 1.85; 95% CI, 1.21–2.84; *p* = 0.005) and DFS (HR 1.95; 95% CI, 1.28–2.97; *p* = 0.002) compared with EAC (Figure 1(a)), while nESCC compared with ESCC patients showed no significant improvement in OS (HR 0.82; 95% CI, 0.78–1.42; *p* = 0.48) and DFS (HR 0.92; 95% CI, 0.52–1.62; *p* = 0.77) (Figure 1(b)).

**Figure 2:**
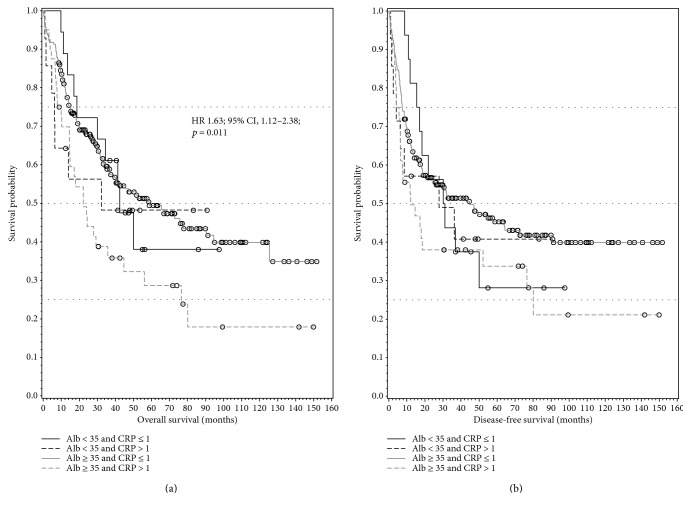
(a) Kaplan-Meier plot showing significantly shorter OS (overall survival) for patients with elevated preoperative CRP value. (b) Kaplan-Meier plot shows a not significant correlation for CRP and/or albumin concerning DFS (disease-free survival).

The interaction of preoperative elevated CRP and preoperative hypoalbuminemia was not statistically significant, indicating that these two effects do not interfere with each other. While patients with preoperative elevated CRP showed a significantly shorter OS (HR 1.63; 95% CI, 1.12–2.38; *p* = 0.011) in simple Cox regression, no significant difference in OS and DFS could be found for patients having preoperative elevated CRP and preoperative hypoalbuminemia (Figures 2(a) and 2(b)).

## 5. Discussion

The present study conducts preoperative prognostic factors and scores, based on serum C-reactive protein and albumin, regarding their predictive value for patients suffering from EC. Today, a multimodal approach using neoadjuvant chemo(radio)therapy followed by radical surgery has been established as the standard treatment strategy for locally advanced EC. To the best of our knowledge, no data is available determining the prognostic value of preoperative C-reactive protein and hypoalbuminemia, GPS, mGPS, and C/A ratio in a patients' collective consisting of EAC, nEAC, ESCC, and nESCC.

Inflammation and cancer are closely related, and there is increasing data that inflammatory cells play a crucial role in cancerogenesis. Cancer is always accompanied by inflammatory processes, creating a tumor microenvironment that leads to tumor angiogenesis, invasion, and metastasis through the recruitment of regulatory T-lymphocytes, activation of cytokines, and the secretion of CRP [13, 14, 40–42]. CRP is an acute phase protein produced in the liver, initially identified appearing in inflammations caused by pneumococcal C-polysaccharide [43]. Elevated levels of CRP are significantly associated with poor survival in various tumor types, including EC [44]. In our study, CRP > 1 mg/dl was significantly associated with shorter OS (*p* = 0.011) in simple analysis. These findings are in good accordance with the data from Huang et al. [45]. However, CRP was not an independent prognostic factor in multiple analyses (*p* = 0.07) in our cohort. Additionally, patients with elevated CRP but normal albumin showed no significantly shorter OS in Kaplan-Meier analysis (Figure 1(b)). Regarding DFS, elevated preoperative CRP was not a prognostic factor, neither in simple or multiple analyses nor in Kaplan-Meier analysis.

Albumin is a central element of plasma proteins conserving the colloidal osmotic pressure and imitates the nutritional status of cancer patients [46].

Malnutrition is common in patients suffering from EC and preoperative hypoalbuminemia is an established prognostic factor for morbidity and mortality in gastro-intestinal cancer patients [47, 48]. Due to accumulation in the tumor tissue, albumin represents an important source of energy and nutrition for the tumor. Beside that nutritional role, serum albumin level represents a marker for inflammatory response in cancer patients as well. The connection of hypoalbuminemia, inflammation, and deprived survival in colorectal cancer patients could previously be shown by Al-Shaiba et al. [49]. In addition, increasing evidence indicates that hypoalbuminemia is strongly connected with poor survival in gastric and esophageal cancer [26, 48, 50]. Especially against that background, it is quite surprising that we did not find significant correlation between hypoalbuminemia and OS (*p* = 0.76) and DFS (*p* = 0.88) in our patients' collective.

More than ten years ago, the Glasgow prognostic score (GPS) and the modified Glasgow prognostic score (mGPS) were the first CRP- and albumin-based prognostic scores introduced [20, 51]. Initially used in advanced stage cancer patients, soon the two scores were used for a number of localized, resectable cancer as well [52–54]. The GPS and the mGPS are simple to calculate and are supposed to reveal the host systemic immune and inflammatory response. Several studies revealed their usefulness as a predictor of survival in patients with various cancers. In addition, recently published data revealed a correlation of the mGPS with the development of tolerance to chemotherapy [55–57]. As we already supposed, knowing the missing significance in the single factors CRP and hypoalbuminemia, no significant correlation in OS and DFS for the GPS (*p* = 0.23 and *p* = 0.76) and the mGPS (*p* = 0.16 and *p* = 0.77) was found in our patients' collective.

Beside the GPS and the mGPS, another CRP and albumin-based prognostic score was introduced recently—the CRP and albumin ratio (C/A ratio). After a study showed that the C/A ratio is an independent risk factor of mortality in septic patients, other studies could show that the C/A ratio is significantly associated with poor outcome of cancer patients [25, 58, 18]. Unfortunately, just like the GPS and the mGPS, no statistical significance could be found for the correlation of C/A ratio and OS (*p* = 0.76) and DFS (*p* = 0.80).

Surgical resection is the cornerstone of curative treatment for esophageal cancer. Besides that, especially in locally advanced cases of EC, multimodal approaches, combining surgery, and neoadjuvant chemo(radio)therapy are increasingly used strategies. Randomized trials, like the CROSS trial, proved significant better outcome in EC patients receiving neoadjuvant chemoradiotherapy [59]. In resectable cancers, including EC, well established prognostic factors like surgical radicality, degree of tumor differentiation, and pathological TNM stage were available after surgery only. Therefore, a number of preoperative available factors like CRP and albumin were investigated and established. Due to the implementation of neoadjuvant treatment in many different solid malignancies, a new moment to evaluate prognostic factors was available. Recently, Shapiro et al. investigated the role of several established pretreatment prognostic factors, such as age, sex, weight loss, and clinical TNM stage in neoadjuvant-treated EC patients [60], but hardly any data is available, evaluating the prognostic role of preoperative levels of CRP and albumin, GPS, mGPS, and C/A ratio in neoadjuvant-treated esophageal cancer. To the best of our knowledge, only one study evaluated the prognostic value of mGPS in relation to neoadjuvant treatment in nESCC and no data for nEAC is published until now. Beside mGPS in simple analysis (*p* = 0.024), we could not find any statistical significant correlation between CRP, albumin, GPS, mGPS, and C/A ratio in our collective, whereas in the study from Otowa et al., the mGPS could be presented as an independent prognostic factor in patients with clinical stage II and stage III nESCC [61].

While looking at all the well performed and published data available, proofing the role of CRP, albumin, GPS, mGPS, and C/A ratio as prognostic factor for many different cancers, we can only speculate why we could not find significance concerning OS and DFS in our collective. Especially the limited number of patients in our subgroups EAC, nEAC, ESCC, and nESCC may be a reason for this finding.

Another potential reason could be differences in the preoperative patient selection. Whereas most of the studies mention that, as a result of the previously published data, they will use the investigated preoperative factors for patients' selection (surgery or conservative therapy), in our center patients showing elevated CRP levels or hypoalbuminemia are initially optimized before performing resection. This preoperative improvement of our patients' general condition might be one of the reasons why the evaluated preoperative laboratory values and scores could not be proved as independent prognostic factors. To survey this hypothesis, it would be necessary to review and compare the median of the preoperative available laboratory values. In case of existing significant differences, this could be an explanation for our missing significance.

As a conclusion, based on our findings, established factors like histology, including tumor size and differentiation, lymph node involvement, and status of resection margin remain the only reliable prognostic factors for patients suffering from resectable EC. To define preoperative available independent prognostic markers, in patients suffering from EC, further investigations are urgently needed.

## Figures and Tables

**Table 1:** Frequency of clinicopathological characteristics.

Variables	*N* (%)
*Age* ^∗^	62.99 ± 10.42
*Gender*	
Male	225 (79.5)
Female	58 (20.5)
*Group of 4*	
EAC	84 (29.7)
nEAC	117 (41.3)
ESCC	32 (11.3)
nESCC	50 (17.7)
*Neoadjuvant treatment*	
Yes	167 (59.0)
No	116 (41.0)
*Adjuvant treatment*	
Yes	30 (10.6)
No	253 (89.4)
*Depth of tumor*	
pT0/ypT0	1 (0.4)/13 (4.6)
pTis/ypTis	3 (1.1)/1 (0.4)
pT1/ypT1	58 (20.5)/17 (6.0)
pT2/ypT2	21 (7.4)/29 (10.2)
pT3/ypT3	25 (8.8)/94 (33.2)
pT4/ypT4	4 (1.4)/8 (2.8)
pTx/ypTx	4 (1.4)/5 (1.8)
*Nodal stage*	
pN0/ypN0	77 (27.2)/70 (24.6)
pN1/ypN1	35 (12.3)/75 (26.5)
pN2/ypN2	1 (0.4)/9 (3.2)
pN3/ypN3	2 (0.8)/13 (4.6)
pNx/ypNx	1 (0.4)/0 (0.0)
*Tumor grading*	
G1	13 (4.6)
G2	125 (44.2)
G3	117 (41.3)
G4	25 (8.8)
Gx	3 (1.1)
*UICC stage*	
0	27 (9.5)
1	75 (26.5)
2	80 (28.3)
3	97 (34.3)
4	4 (1.4)
*Surgical approach*	
One-stage	233 (82.3)
Two-stage	50 (17.7)

^∗^Age is reported as the mean ± SD. EAC: esophageal adenocarcinoma; nEAC: neoadjuvant-treated esophageal cancer; ESCC: esophageal squamous cell carcinoma; nESCC: neoadjuvant-treated esophageal squamous cell carcinoma; UICC: Union for International Cancer Control.

**Table 2:** Details for EC subgroups.

Variable	EAC *N* (%)	nEAC *N* (%)	ESCC *N* (%)	nESCC *N* (%)
*Gender*				
Male	68 (81.0)	100 (85.5)	21 (65.6)	36 (72.0)
Female	16 (19.0)	17 (14.5)	11 (34.4)	14 (28.0)
*UICC stage*				
0	6 (7.1)	8 (6.8)	2 (6.3)	11 (22.0)
1	51 (60.7)	12 (10.3)	7 (21.9)	5 (10.0)
2	17 (20.2)	35 (29.9)	11 (34.4)	17 (34.0)
3	9 (10.7)	59 (50.4)	12 (37.5)	17 (34.0)
4	1 (1.2)	3 (2.6)	0 (0.0)	0 (0.0)
*Adjuvant therapy*				
Yes	1 (1.2)	24 (20.5)	2 (6.3)	3 (6.0)
No	83 (98.8)	93 (79.5)	30 (93.7)	47 (94.0)
*Surgical approach*				
One-stage	29 (34.5)	20 (17.1)	0 (0.0)	1 (2.0)
Two-stage	55 (65.5)	97 (82.9)	32 (100)	49 (98.0)
*CRP > 1* ^∗^				
Yes	11 (13.3)	19 (16.7)	7 (21.9)	17 (34.0)
No	72 (86.7)	95 (83.3)	25 (78.1)	33 (66.0)
*Alb < 35* ^∗^				
Yes	10 (12.0)	14 (12.2)	1 (3.1)	7 (14.0)
No	73 (88.0)	101 (87.8)	31 (96.9)	43 (86.0)
*GPS* ^∗^				
0	64 (77.1)	86 (75.4)	25 (78.1)	32 (64.0)
1	17 (20.5)	23 (20.2)	6 (18.8)	12 (24.0)
2	2 (2.4)	5 (4.4)	1 (3.1)	6 (12.0)
*mGPS* ^∗^				
0	72 (86.8)	95 (83.3)	25 (78.1)	33 (66.0)
1	9 (10.8)	14 (12.3)	6 (18.8)	11 (22.0)
2	2 (2.4)	5 (4.4)	1 (3.1)	6 (12.0)
*C/A ratio > 0.95* ^∗^				
Yes	4 (4.8)	6 (5.3)	1 (3.1)	6 (12.0)
No	79 (95.2)	108 (94.7)	31 (96.9)	44 (88.0)

^∗^In four patients, preoperative CRP and/or albumin was not available. EC: esophageal cancer; EAC: esophageal adenocarcinoma; nEAC: neoadjuvant-treated esophageal cancer; ESCC: esophageal squamous cell carcinoma; nESCC: neoadjuvant-treated esophageal squamous cell carcinoma; UICC: Union for International Cancer Control; CRP > 1: preoperative serum C-reactive protein >1 mg/dl; Alb < 35: preoperative serum albumin <35 g/l; GPS: glasgow prognostic score; mGPS: modified Glasgow prognostic score; C/A ratio: ratio from preoperative serum CRP and albumin.

**Table 3:** Results of simple and multiple Cox regression models for overall survival. Hazard ratios (HR) with 95% confidence intervals (CI) and *p* values (*p*) and proportions of explained variation (PEV). Full model PEV = 21.8%.

Variables	Simple HR (95%, CI)	*p* value	PEV	Multiple HR (95%, CI)	*p* value	PEV
Alb < 35	1.01 (0.61–1.68)	0.97	0.0	0.92 (0.54–1.58)	0.763	0.0
CRP > 1	1.63 (1.12–2.38)	0.011	1.6	1.47 (0.97–2.24)	0.070	0.3
N1^∗^	3.42 (2.01–5.82)	<0.001	12.4	1.93 (0.98–3.77)	0.007	3.1
N2^∗∗^	3.13 (2.21–4.43)	<0.001	12.4	2.37 (1.50–3.74)	<0.001	3.1
Nres^∗∗∗^	0.90 (0.77–1.05)	0.18	0.3	0.70 (0.59–0.83)	<0.001	3.2
nEAC^∗∗∗∗^	1.85 (1.21–2.84)	0.005	4.2	1.03 (0.62–1.72)	0.903	1.2
nESCC^∗∗∗∗^	0.82 (0.78–1.42)	0.482	4.2	0.85 (0.47–1.53)	0.589	1.2
One-stage surgery	0.56 (0.33–0.94)	0.027	1.5	0.80 (0.46–1.41)	0.447	0.1
UICC		<0.001	13.5		0.013	3.0
I versus 0	0.59 (0.26–1.30)	0.186		0.77 (0.33–1.82)	0.555	
II versus 0	2.03 (1.00–4.13)	0.052		1.94 (0.89–4.21)	0.093	
III + IV versus 0	2.90 (1.45–5.84)	0.003		2.58 (1.08–6.14)	0.033	

^∗^Effect of at least one pos. LK has time-dependent effect (*p* = 0.001 in simple model, *p* = 0.002 in multiple model). HR and 95% CI given are evaluated at 48 months; *p* value given refers to whole (time-dependent) effect, including neoadjuvant-treated and untreated cases. ^∗∗^N1 and N2 together also in “simple” model, including neoadjuvant-treated and untreated cases. ^∗∗∗^HR for log2-transformed variables quantify the effect of a doubling of the respective variable. ^∗∗∗∗^Neoadjuvant therapy and tumor biology (EAC and ESCC) exhibit a significant interaction (*p* = 0.021) in the model containing only these two variables but not in the multivariable model (*p* = 0.604). Thus, the neoadjuvant therapy effect is given separately for nEAC and nESCC.

**Table 4:** Multiple Cox regression models for overall survival for various proposed ways to account for CRP and albumin effect. Results for multiple Cox regression model refer to adjustment for the variables reported in Table 3. Hazard ratios (HR) with 95% confidence intervals (CI) and *p* values (*p*) and proportions of explained variation (PEV). Full model PEV = 21.8%.

Variables	Multiple HR (95%, CI)	*p* value	PEV
Alb < 35	0.92 (0.54–1.58)	0.763	0.0
CRP > 1	1.47 (0.97–2.24)	0.070	0.3
GPS		0.287	0.3
1 versus 0	1.37 (0.93–2.04)	0.116	
2 versus 0	1.17 (0.52–2.60)	0.708	
mGPS		0.159	0.3
1 versus 0	1.55 (0.99–2.43)	0.056	
2 versus 0	1.17 (0.53–2.60)	0.705	
log(CRP/Alb)^∗^	1.02 (0.95–1.09)	0.684	0.0
CRP/Alb > 0.95	0.89 (0.44–1.84)	0.759	0.0

^∗^HR for log2-transformed variables quantify the effect of a doubling of the respective variable.

**Table 5:** Results of simple and multiple Cox regression models for disease-free survival. Hazard ratios (HR) with 95% confidence intervals (CI) and *p* values (*p*) and proportions of explained variation (PEV). Full model PEV = 23.4%.

Variables	Simple HR (95%, CI)	*p* value	PEV	Multiple HR (95%, CI)	*p* value	PEV
Alb < 35	1.08 (0.67–1.75)	0.75	0.0	0.96 (0.57–1.61)	0.878	<0.1
CRP > 1	1.44 (0.98–2.12)	0.066	0.9	1.10 (0.72–1.70)	0.648	<0.1
N1^∗^	2.04 (1.33–3.13)	0.001	15.0	1.01 (0.56–1.82)	0.981	3.9
N2^∗^	2.29 (1.50–2.50)	<0.001		2.58 (1.63–4.10)	<0.001	
Nres^∗∗^	0.97 (0.83–1.15)	0.741	0.0	0.78 (0.65–0.93)	0.005	1.9
nEAC^∗∗∗^	1.95 (1.28–2.97)	0.002	4.2	0.95 (0.56–1.59)	0.836	1.6
nESCC^∗∗∗^	0.92 (0.52–1.62)	0.767		0.87 (0.47–1.62)	0.668	1.6
One-stage surgery	0.67 (0.42–1.07)	0.094	0.9	0.97 (0.58–1.61)	0.906	0.0
UICC		<0.001	15.4		0.002	3.5
I versus 0	0.66 (0.30–1.44)	0.293		0.86 (0.37–1.99)	0.718	
II versus 0	2.08 (1.02–4.26)	0.045		2.27 (1.04–4.95)	0.04	
III + IV versus 0	3.54 (1.76–7.10)	<0.001		3.43 (1.42–8.33)	0.006	

^∗^N1 and N2 together also in “simple” model. ^∗∗^HR for log2-transformed variables quantify the effect of a doubling of the respective variable. ^∗∗∗^Neoadjuvant therapy and EAC and ESCC exhibit a significant interaction (*p* = 0.037) in the model containing only these two variables but not in the multivariable model (*p* = 0.834). Thus, the neoadjuvant therapy effect is given separately for EAC and ESCC.

**Table 6:** Multiple Cox regression models for disease-free survival for various proposed ways to account for CRP and albumin effect. Results for multiple Cox regression model refer to adjustment for the variables reported in Table 3. Hazard ratios (HR) with 95% confidence intervals (CI) and *p* values (*p*) and proportions of explained variation (PEV).

Variables	Multiple HR (95%, CI)	*p* value	PEV
Alb < 35	0.96 (0.57–1.61)	0.878	<0.1
CRP > 1	1.10 (0.72–1.70)	0.648	<0.1
GPS		0.758	0.0
1 versus 0	1.15 (0.77–1.71)	0.494	
2 versus 0	0.93 (0.44–1.96)	0.84	
mGPS		0.768	<0.1
1 versus 0	1.17 (0.73–1.87)	0.507	
2 versus 0	0.92 (0.44–1.94)	0.825	
log(CRP/Alb)^∗^	1.00 (0.94–1.08)	0.93	0.0
CRP/Alb >0.95	0.91 (0.46–1.81)	0.79	0.0

^∗^HR for log2-transformed variables quantify the effect of a doubling of the respective variable.
